# Does Educational Attainment Affect Residents’ Health?

**DOI:** 10.3390/healthcare8040364

**Published:** 2020-09-24

**Authors:** Cuihong Long, Pei Liu, Chengzhi Yi

**Affiliations:** 1 School of Economics, East China Normal University, Shanghai 200062, China; chlong@jjx.ecnu.edu.cn (C.L.); 51184401008@stu.ecnu.edu.cn (P.L.); 2 School of International and Public Affairs, China Institute for Urban Governance, Shanghai Jiaotong University, Shanghai 200030, China

**Keywords:** educational attainment, health returns, structural equation model

## Abstract

Based on the data of the Chinese General Social Survey 2015 (CGSS2015), this article conducts an empirical analysis on the relationship between education and health status of Chinese residents by using the structural equation model (SEM), the propensity score matching (PSM) method, and generalized ordered logit (Gologit) model. Our study found that education promotes both the subjective and objective health of residents, and the finding holds true after considering the selection bias. In addition to having a direct role, education could promote health through improved mental health, economic status, and healthy behaviors. The finding is consistent with the explanations in existing research of “efficiency-improving effect”, “mental health effect”, and “budget relaxation effect”. Further research on the mechanism of education affecting health through structural equation modeling finds that mental health plays a more important role than healthy behaviors and economic status. In terms of the differences of various groups, education has stronger effect on vulnerable groups with fewer social resources, which shows that education helps reduce health inequality. The conclusion has important policy significance.

## 1. Introduction

Individual health is the foundation of the healthy development of the economy and society. What factors affect individual health has long been a topic of concern for researchers [1,2,3,4,5,6,7]. The studies have promoted the understanding of the factors affecting individual health. However, the relationship between education and health is not yet clearly understood.

In this context, studying the relationship between education and health has important significance. First, existing research has mainly focused on developed countries, and the research on developing countries is insufficient. Because countries with different levels of development have great differences in economic development, health systems, and education policies, education might play a different role in the health of residents in different countries [8]. As the most populous developing country in the world, China’s economy has been developing rapidly since the reform and opening up. People’s living standards have also improved, and great attention is directed to physical and mental health and the quality of life. However, there are still a series of challenges facing the health of residents in China. Relevant data show that the deaths caused by chronic noncommunicable diseases, such as cardiovascular and cerebrovascular diseases, cancer, chronic respiratory diseases, diabetes, and other diseases, account for 88% of total deaths [9]. In the report of the 19th National Congress of the Communist Party of China in 2017, the “Healthy China Strategy” was clearly proposed, and the health of residents was elevated to a national strategy. Research on the relationship between education and health not only helps provide specific policy tools for the implementation of the healthy China strategy but also provides valuable insights for advancing research on education and health in similar countries. Second, the existing research offers much discussion on the relevance of education and health, but it lacks an in-depth analysis of the relationship between the two. Through what channels does education affect health? What are the direct and indirect effects? What is the relative strength of various channels’ effect on health? These questions have not yet been discussed in detail. Last, the existing literature still needs to analyze the differences in the role of education for different groups. The heterogeneity analysis of the health returns of education to different groups in this article could help scholars better understand the influence of education and make relevant suggestions in a more targeted manner.

The purpose of this article is to empirically test whether and how education affects the health of residents based on a large national sample in China. The study found that education has a positive effect on both the subjective and objective health of residents. The marginal contributions of this article are as follows: First, this article takes Chinese residents’ educational health returns as the research object, which is a valuable supplement to the samples of developing countries. Second, by constructing a structural equation model, this article deeply analyzes the influence of education on health, the direct and indirect effects, and the relative strength of various pathways and reveals the health returns of education in more detail. Last, this article analyzes the heterogeneity of educational health returns from multiple dimensions and discusses the differences in gender, age, urban–rural residence, and regional educational health returns, revealing the “resource substitution effect”. Resource substitution effect means resource will play a greater role in groups owning fewer other resources. In this article, it is mainly reflected in the higher health returns of education for rural residents and female groups with fewer social and economic resources. Our study provides helpful reference for the formulation of public health, education, and other policies.

The rest of this article is structured as follows: The second part is the literature review and hypothesis. The third part introduces the materials and methods. The fourth part analyzes the empirical results. Finally, the research conclusion is drawn in the fifth part.

## 2. Materials and Methods

### 2.1. Literature Review

#### 2.1.1. Research on Influencing Factors of Health

The existing literature on health-influencing factors primarily offers analysis at the structural level and the individual level. For example, the structural level mainly focuses on medical and health services [1,10] and environmental quality [6], while the individual level mainly includes income level [2,3,11], social capital [4,7], and education level [5,12,13,14], although the distinction between the two levels is blurred. 

The impact of income on health is considered based on the absolute income hypothesis [2,5,11] and the relative income hypothesis [15,16,17,18]. In terms of absolute income, a large number of studies have proven that there is a “health-income stratification” phenomenon between income and health. By improving nutrition, medical investment, lifestyle habits, healthy behaviors, and other factors, high-income groups might have better physical and mental health, longer life expectancy, and lower mortality rates than low-income groups [3,11,19]. The impact of relative income on health, mainly focuses on the effect of the equity of income distribution on health. Most studies have shown that income gaps are not conducive to the health of individuals and society as a whole [15,16,17].

Medical and health services, including the development of medical technology and public health expenditure policies, such as medical insurance, are believed to be critical to the overall health of residents in a country. Advances in medical technology largely explain the increase in life expectancy of a country’s residents [11]. Expansion of public health expenditure could increase the supply of medical facilities and staff, expand the supply of preventive health services, and increase the access of patients to medical treatment, while medical insurance policy could reduce the cost of medical treatment for patients, increase their purchasing power of medical services to receive timely and effective treatment, and thus promote personal health [1,10,20]. The research by Bidani et al. shows that public health expenditures in a country could effectively increase the life expectancy of residents, with a more significant effect on low-income groups [20]. Flegg’s study of 46 underdeveloped countries found that an increase in the number of doctors and nurses could significantly reduce infant mortality [21].

Social capital is believed to strengthen the mutual trust among residents, enabling individuals to obtain material and emotional help and support, and reduce psychological pressure when individuals face difficulties, thereby contributing to physical and mental health [4,22]. Yip et al.’s research on the health status of rural residents in China shows that social trust is positively related to personal health, mental health, and well-being [7]. Lindstr et al.’s study of Sweden also proved the positive effect of social capital on self-assessed health [23]. Franzini et al.’s research on Texas in the United States found that social capital could effectively reduce the premature death of heart disease patients [24].

According to the health demand model and related research proposed by Grossman [5,12,13], education has a strong positive correlation with individual health. The impact of education on individual health is discussed in detail in the next section.

#### 2.1.2. Research on the Relationship between Education and Health

The existing research on the relationship between education and health mainly discusses two aspects of the problem. First, scholars examine whether education would improve individual health and the overall mechanism [14,25]. This type of research has not yet reached a unified conclusion. Some scholars believe that a higher education level would improve the health of residents [5,12,13,14,25]. The second kind of research focuses on a certain group and studies the specific impact of education on the group and the mechanisms [8,26,27], which is also understudied. The existing literature mainly focuses on the elderly and migrant workers [27,28,29]. Cheng et al. used Chinese Longitudinal Healthy Longevity Survey (CLHLS) data to study the effect of education on the health of the elderly, and the results showed that education has a significant role in promoting the health of the elderly [28]. Kye et al. studied the relationship between the health of the elderly in South Korea and their education level and found that education could effectively improve the health of the elderly and their offspring [27]. It can be seen that despite the differences in opinion, most researchers believe that education helps improve the health level of residents.

If education has an impact on the health of residents, how does this effect occur? The existing literature posits that education could affect residents’ health in the following ways:

One is the “budget relaxation effect”; that is, groups with higher education levels can often obtain better occupations and higher incomes so that more funds can be invested in personal health, such as regular medical examinations and insurance [30,31]. Generally, with the improvement of academic qualifications, individuals’ job ranks and economic incomes tend to increase. As a result, personal health care and investment increase accordingly, with a positive effect on promoting residents’ health. Based on this, we propose the following research hypothesis:

**Hypothesis** **1** **(H1).** *Educational attainment promotes residents’ health by raising their income.*

The second channel is the “efficiency-improving effect”, which means that the same health investment would yield a higher output to the group with higher education than to the group with lower education. This may be because more educated people have more knowledge about physical and mental health. They tend to pay more attention to personal health care, maintain good living habits, adopt more scientific treatment methods and actively cooperate with doctors’ treatment schemes when they become ill [14,32]. Generally, with the improvement of the education level of residents, personal physical and mental health knowledge expand. It is then easier to maintain good habits and cultivate a positive and optimistic attitude towards life, which is of great benefit to physical and mental health. Based on this, we propose the following research hypothesis:

**Hypothesis** **2** **(H2).** *Educational attainment improves the health of residents by promoting healthy behaviors.*

The third channel is the “mental health effect”; that is, people with higher education levels pay more attention to mental health and spiritual life. When they encounter setbacks or high mental stress, they can find a positive way to resolve negative emotions and maintain a positive and optimistic attitude towards life [29]. However, some scholars have found that education has no obvious role in promoting health [33,34]. Therefore, whether the mental health effect exists still needs to be tested. Here, this article proposes the following research hypothesis based on mental health effects:

**Hypothesis** **3** **(H3).** *Educational attainment can promote the health of residents by improving their mental health.*

### 2.2. Data, Variables and Methods

#### 2.2.1. Data Source

The data for this study come from the 2015 Chinese General Social Survey, which was jointly conducted by Renmin University of China and academic institutions across the country. In 2015, the investigation team conducted a sample survey covering 10,968 households in 28 provinces, autonomous regions, and municipalities in mainland China. After excluding samples with missing values that were not suitable for this study, we obtained 8680 samples, which were representative.

#### 2.2.2. Variable Design

##### Dependent Variables

The dependent variables in this article include a subjective health evaluation and an objective health status. Subjective health evaluation is considered an effective indicator to measure health and has been widely used in research [35]. It is measured by the 5-point Likert scale in the CGSS2015 questionnaire. The corresponding question is, “What do you think of your current physical health?”. Based on the respondents’ answers, we constructed an ordinal variable consisting of 5 levels, in which very unhealthy is assigned a value of 1 and very healthy a value of 5. The objective health status is measured by the question, “The frequency at which work was affected by health in the past four weeks”, in which never is assigned a value of 1, rarely a value of 2, sometimes a value of 3, frequently a value of 4, and always a value of 5.

##### Independent Variables

The key independent variable in this article is the education level of the interviewee. The corresponding question in the questionnaire is, “What is your highest level of education?”. According to the different levels of education from low to high, no education is assigned a value of 1, old-style private school, literacy classes, and primary school are classified as “literacy to primary school” and are assigned a value of 2, and junior high school is assigned a value of 3. Vocational school, technical school, and senior high school are classified as “senior high school and equivalent” and are assigned a value of 4, college (formal higher education/adult higher education) a value of 5, and undergraduate (formal higher education/adult higher education) a value of 6. Graduate education and above are assigned a value of 7.

##### Control Variables

Referring to the existing research, the control variables in this article include parents’ education level, gender, age, ethnicity, political status, marital status, region, etc. For the education level of parents, we adopt a coding method similar to that applied to the education level of the respondents. Gender is a dichotomous variable, where female is assigned a value of 0 and male a value of 1. Age measures the actual age of the respondent. Political status is a dummy variable in which Chinese Communist Party (CCP) member is assigned a value of 1, and others the value 0. Ethnicity is a dummy variable, where the Han is assigned a value of 1, and others the value 0. Marital status is also a dummy variable, where married is assigned a value of 1; otherwise, 0. In addition, due to the differences in social development, economic conditions, environmental quality, and other factors in different regions, people living in different places may have divergent health levels. To control such regional divergences, this article introduces province dummy variables to reduce the evaluation bias that may be caused by various regions.

In addition, according to the theoretical analysis of this article, we control the three variables of budget constraint relaxation, healthy lifestyle, and mental health.

Budget constraint relaxation is measured by three indicators of horizontal economic status, vertical economic status and family economic strength [28]. (1) Horizontal economic status is measured by the respondents’ economic status compared with their peers, where lower status is assigned a value of 1, and higher status is assigned a value of 3. (2) Vertical economic status is measured by the respondents’ economic status compared with their own situation three years ago, where decline is assigned a value of 1, similarity a value of 2, and improvement a value of 3. (3) Family economic strength is measured by the level of the family’s economic status. We use the 5-point Likert scale to assign a value of 1 to far below the average level and 5 to far above the average level.

Healthy lifestyle is measured by the three indicators of the frequency of physical exercise, learning new knowledge, and listening to music [28,36,37]. (1) The frequency of physical exercise is measured by the 5-point Likert scale, where 1 represents never and 5 represents every day. (2) The frequency of learning new knowledge and listening to music is measured in the same way. (3) The frequency of learning new knowledge is measured by the 5-point Likert scale, where 1 means never and 5 means very frequent.

Mental health is also measured by three indicators: the sense of happiness, the frequency of depression, and social trust [3,7,38]. Social trust refers to individual’s trust in people around him, including neighbors, colleagues, friends, and the whole society. People with strong social trust think that most people are trustworthy and have a sense of inner security. They tend to have a positive and optimistic attitude to life. While people with low social trust are unwilling to trust others and lack of inner security, which may adversely affect mental health. (1) The 5-point Likert scale is used for the measurement of happiness, where very unhappy is assigned a value of 1, and very happy is 5. (2) The frequency of depression in the past four weeks is measured by a 5-point Likert scale, where 1 means never and 5 means always. (3) Social trust is also measured by a 5-point Likert scale, where 1 represents very distrustful and 5 represents very trustful.

Table 1 shows the descriptive statistical results of the variables. The interviewees were generally healthy, and their work was affected by health at a lower frequency. The average education level of the interviewees was between junior high school and senior high school, the average age was approximately 55 years old, and the gender distribution was basically balanced. The education of parents was mostly at the elementary level. The frequency of respondents’ learning new knowledge was generally low, while physical exercise and listening to music approached several times a month. Respondents felt low depression and had a higher sense of happiness and social trust. In terms of economic status, personal and family economic status were at an average level for the majority of the respondents, while their vertical economic status was similar to their situation three years ago.

#### 2.2.3. Methods

According to the assumptions above, we establish the following basic model:

$$H_{i}=\alpha Education_{i}+\beta X_{i}+\gamma C_{i}+\varepsilon_{i}$$

Among them, $H_{i}$ represents interviewee i’s evaluation of his own health. $X_{i}$ represents a series of control variables. $C_{i}$ represents the three channel variables of budget constraint relaxation, healthy lifestyle, and mental health, and $\varepsilon_{i}$ is a disturbance item. In terms of coefficients, *α* represents the regression coefficient of individual education to health, *β* represents that of control variables to individual health, and *γ* represents that of channel variables to individual health.

On this basis, we will use the propensity score matching method, the SEM method and generalized ordered logit model to analyze the relationship between education level and health status of residents. Since there are factors that might affect both the level of education and health, the problem of selection bias should be considered. We will use the PSM method to address the potential problem existing in the model. In order to explore how education affects health, we will use the SEM method, which is a method much favored by scholars because it can estimate the relationship between multiple interrelated variables in a single analysis. It has been widely used in social science and interdisciplinary research. In addition, we will also use the generalized ordered logit model for robustness test.

## 3. Results

### 3.1. The Relationship between Education Level and Health

First, we use the Ordinary Least Squares (OLS) method to perform a basic regression on the model and gradually add control variables on the basis of the core explanatory variable, education, to initially investigate the relationship between education level and health status. Model 1 only adds the explanatory variable of the education level, Model 2 adds age and gender on the basis of Model 1, and Model 3 adds parents’ education on the basis of Model 2. All models control for provincial dummy variables, and the regression results are shown in Table 2.

It can be seen from Table 2 that education level has a positive correlation with self-reported health and the objective health level, both of which are significant at the level of 1%. Specifically, the higher the level of education is, the higher the respondents’ self-reported health and the lower the frequency of health effects on work. This is consistent with existing research findings [25,39]. There is a significant negative correlation between age and health status. The older the age, the lower the subjective health assessment and the objective health level. Gender is significantly and positively correlated with health. Whether subjective health evaluation or objective health, men have better health than women. One possible explanation could be that, compared with men, women generally devote more time and energy to caring for their children and doing housework. At the same time, they are also expected to handle job affairs well. Thus, women often face more difficulties in balancing family responsibility and career development, which might affect their health status both subjectively and physically. Parents’ education level is significantly positively related to their children’s subjective and objective health status, while the role of mothers is more important than that of fathers. For each increase in the level of mother’s education, the children’s self-reported health will improve 0.033 grades, which is 0.006 grades higher than the effect of father’s education, and the frequency of work affected by health will decrease 0.031 grades, which is 0.004 grades lower than the effect of father’s education. Both of them are significant at the level of 1%.

This is consistent with the findings of Currie et al. and Case et al. [40,41]. One possible explanation is that parents’ education level largely determines children’s family background and growth environment. If parents are well educated, on the one hand, they will follow and guide their children’s growth in a more scientific and reasonable manner, while on the other hand, they will also give their children a better economic foundation to meet nutritional and material needs so that their children can be healthier. The mother’s more important role may be because in the traditional division of labour in Chinese families, the father mainly bears the financial responsibility, and the mother mostly plays the role of assisting the husband and bringing up children, investing more time and energy into caring for and accompanying children. The mother’s higher education level can enable her to give her children more scientifically based and comprehensive care and guidance, thus helping her play a greater role in the health of her children.

### 3.2. Discussion on the Issue of Selection Bias

In addition to education level, factors such as childhood growth environment, family background, gender, and age may affect health [41,42]. These factors might also affect the access to education and the education level of a person [43,44]. For example, some individuals might have a lower level of education due to low fa0mily income. At the same time, because of low family income, they might have less access to health resources and health care services, which could affect their health status. As these individual and family-level characteristics could affect both access to education and health status, they might cause confusion. Thus, there might exist the potential problem of selection bias and PSM method is used to address this problem.

Since the PSM method requires that the treatment variable is a binary variable, we re-code the education level of the respondents, taking nine-year compulsory education as the demarcation point to reclassify the data, in which education levels equal to and lower than primary school level are classified as not receiving complete compulsory education and assigned a value 0, while those equal to and higher than junior high school level are classified as receiving complete compulsory education and assigned a value of 1. According to Chinese law, every citizen has the right and obligation to receive nine years of compulsory education, while education beyond nine years is non-compulsory. Therefore, it is might be reasonable to take nine-year compulsory education as the demarcation point. In this way, we obtain a binary variable of education as the treatment variable.

Table 3 lists the four matching methods and their treatment effects. Among the different matching methods, the matching treatment effect of self-reported health is approximately 0.2, while the frequency of health effects on work is approximately −0.2 (To save space, we did not report the coefficients of covariates after the appropriate PSM adjustment. Readers who need please contact us to obtain the results). This shows that the model has good robustness, not depending on the choice of specific methods, and each matching method is significant at the 1% level. The average treatment effect of self-reported health is 0.209, and the average treatment effect of the frequency of health effects on work is −0.209. This result shows that compared with respondents who have not received a complete compulsory education, those who have finished compulsory education obtain higher self-reported health by an average of 0.209 grades with a one-year increase in education, and the frequency of health effects on work decreases by 0.209 grades, indicating that education has a significant role in promoting individuals’ subjective and objective health.

Since the method of PSM implies a data balancing assumption, that is, the confounding variable no longer significantly affects the treatment variable after matching, we conduct the data balancing test. According to Rosenbaum and Rubin [45], if the absolute value of the standard deviation of the variable after matching is less than 20, then the matching method is appropriate, and the estimated result of the matching is relatively reliable. Table 4 shows that compared with the results before matching, the standard deviations of most variables are greatly reduced, and the standard deviations of the confounding variables are less than 20% after matching. The data balancing assumption can be satisfied.

### 3.3. How Education Affects Health

The above analysis shows that education can indeed promote the health status of residents. After considering the problem of selection bias, the effect is still significant, but how education promotes the health of residents is not yet clear. To answer this question, the SEM method is used.

Based on the above theoretical analysis, we test whether education could improve the health of residents through higher economic status, healthier lifestyles, and better mental health. To this end, we first construct the observation variable of education and three exogenous latent variables: healthy behavior, mental health, and economic status. Healthy behavior is constructed by the three observation variables of the frequency of physical exercise, learning new knowledge, and listening to music. Mental health is constructed by the three observation variables of the sense of happiness, the frequency of depression, and social trust. Economic status is constructed by horizontal economic status, vertical economic status, and family economic status. Moreover, the health status, constructed by self-reported health and the frequency of health effects on work, is set as an endogenous latent variable, which could be affected by healthy behavior, mental health, and economic status.

We used Amos 25.0 to conduct structural equation modeling. IBM SPSS Amos (IBM, Armonk, NY, USA) is a useful software developed by IBM that supports the use of structural equation modeling to test hypotheses about complex variable relationships. According to the reported result of AMOS 25.0 shown in Table 5, the absolute fit index of the model is 0.067, which is less than the critical value of 0.08. In terms of the values for the relative fit index, the comparative fit index (CFI), the normed fit index (NFI), and the increment fit index (IFI) are 0.913, 0.911, and 0.913, respectively, which are all more than the critical value of 0.9. In terms of the values of the simple fit index, the parsimonious goodness-of-fit index (PGFI), the parsimony normed fit (PNFI), and the parsimony comparative of fit (PCFI) are 0.593, 0.662, and 0.664, respectively, which are all more than the critical value of 0.5. However, the ratio of the chi-square value to the degree of freedom is 39.727, which is more than the critical value of 2. This may be due to the large sample size in this article. In the case of a large sample, a significant chi-square value is usually generated. The chi-square degree of freedom ratio is far from meeting the required level. In this case, it is necessary to comprehensively consider other indicators to determine whether the model is suitable [46]. Therefore, combined with indicators of absolute fit index, relative fit index, simple fit index and the sample size, the ratio of the chi-square value to the degree of freedom is acceptable, and the fitting result of the structural equation model is suitable on the whole.

**Figure 1:**
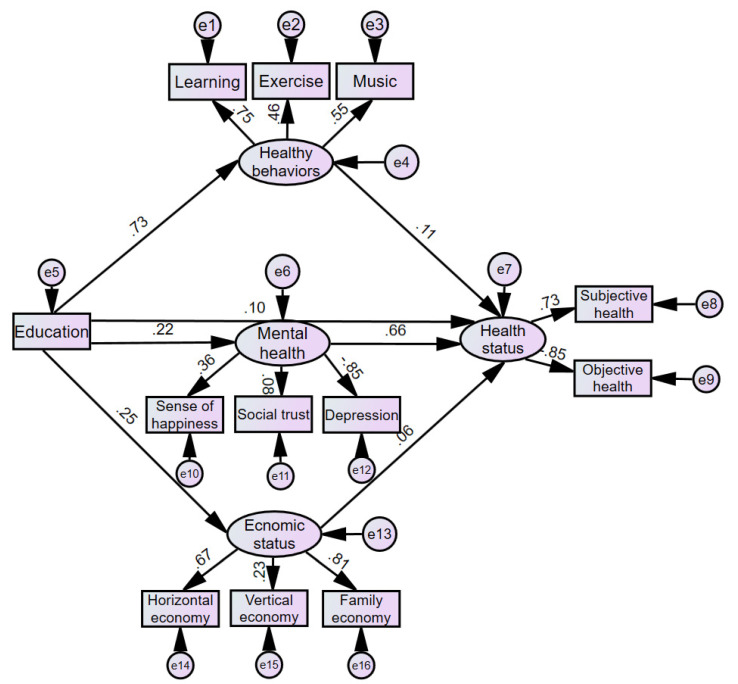
The impact mechanism of education on health. Note: In the figure, the e (e4, e6, e7, e13) with the arrow pointing to the latent variable is the unexplainable part of the regression equation, that is, the residual term, and the e with the arrow pointing to the explicit variable (e1–3, e5, e8–9, e10–e12, e14–e16) is the measurement error; the 0 before the decimal point is omitted.

As shown in Table 6 and Figure 1, the standardized regression coefficient of education level to healthy behavior is 0.73, and the standardized regression coefficient of healthy behavior to health status is 0.11, both of which are significant at the level of 1%, so the mediating effect of education on health status through healthy behavior is 0.73 × 0.11 = 0.08. This shows that education can indeed lead residents to choose a healthier lifestyle and develop better living habits, thereby improving their health. This is consistent with the explanation of “efficiency-improving effect” and further validates the conclusions of Kenkel, Culter and Lleras-Muney, and Cheng [14,28,47]. Specifically, the standardized regression coefficient for healthy behavior and learning new knowledge is the highest, at 0.75, followed by listening to music at 0.55, and physical exercise is relatively low at 0.46, indicating that education has a more significant role in leading residents to learn and listen to music. One possible explanation is that education attainment could affect residents’ interest in learning and listening to music. Thus, residents with a higher level of education might learn and listen to music more as they tend to be more interested in learning and listening to music, which help to obtain spiritual improvement and relaxation, maintain a happy mental state, and thereby promote physical and mental health.

The standardized regression coefficient of education level on mental health is 0.22, while the standardized regression coefficient of mental health on health status is 0.66, both of which are significant at the level of 1%, so the mediating effect of education on health status through mental health is 0.22 × 0.65 = 0.15. This shows that education can improve the mental health of residents and lead residents to maintain a good mentality and a positive attitude towards life. This is consistent with the explanation of “mental health effect”. Specifically, the standardized regression coefficient for mental health and the frequency of depression is the highest, at −0.85, the sense of happiness is 0.36, and social trust is the lowest at 0.08. This shows that the improvement of education level could reduce the frequency of depression and increase the sense of well-being, thus helping residents maintain a good mental state and an improved level of mental health, but it has a relatively weak effect on enhancing social trust or the sense of security.

The standardized regression coefficient of education level to economic status is 0.25, while the standardized regression coefficient of economic status to health status is 0.06, both of which are significant at the level of 1%, so the mediating effect of education on health through economic status is 0.25 × 0.06 = 0.02. This shows that the improvement in education level enables residents to obtain higher income, improve material conditions and living standards, and invest more resources in personal health and care, thereby promoting their health. This is consistent with the explanation of an “efficiency-improving effect” and further validates the conclusions of the existing literature [29,30,31]. In terms of specific indicators of economic status, the standardized regression coefficients of family economic status and horizontal economic status are relatively high, at 0.81 and 0.67, respectively, and the coefficient of vertical economic status is relatively low, at 0.23, indicating that education mainly promotes personal health by improving family economic status and horizontal economic status. This is also easy to understand because the horizontal economic status is easy to perceive in comparison with others, so the increase in economic status among peers often brings more satisfaction and the sense of economic gain. The improvement of family economic status could allow each family member to benefit more and enjoy better living conditions, thereby bringing more health returns.

The standardized regression coefficient of education level on health status is 0.10; that is, the direct effect of education on health is 0.10, and it is significant at the level of 1%. Coupled with the indirect effect of education on health status through healthy behavior, 0.08, the indirect effect through mental health, 0.15, and the indirect effect through economic status, 0.02, the total effect of education on health status is 0.35; that is, for each unit increase in education level, an individual’s health situation will improve on average by 0.35 units. The main reason why the indirect effect of mental health is greater than the effects of healthy behavior and economic status may be that education improves the individual’s economic ability, but it also brings a heavier workload. A common phenomenon is that high-education groups work longer hours and stay up late more frequently, thereby reducing the time for personal leisure, physical exercise, and other healthy activities, which may offset part of the positive effects brought about by the improvement of economic status and healthy behavior. Scholars also found that the sleep hours of high-education groups differed greatly between rest days and workdays.

### 3.4. Heterogeneity Analysis and Robustness Test

#### 3.4.1. Heterogeneity Analysis of the Effect of Education on Health

Through the above analysis, we have confirmed that education affects residents’ health in three ways. However, is there any difference in the role of education in promoting health for different groups? In this section, we analyze this issue.

##### Urban–Rural Differences in Education’s Effect on Health

As shown in Table 7, the health promotion effect of education for rural residents is significantly greater than that for urban residents. Specifically, for each increase in the level of education, the increase in the self-reported health of rural residents is 0.047 grades higher than that of urban residents, and the frequency of health effects on work is 0.057 grades lower than that of urban residents; these effects are significant at the level of 1%. This might be explained by the resource substitution theory of Ross et al. [48]; that is, a resource will play a greater role in groups owning fewer other resources. Due to the long existence of China’s dual structure, the gap between urban and rural development is relatively large. Rural residents generally lag behind urban residents in medical resources, social insurance, and individual economic conditions. Therefore, they might make full use of educational resources and rely more on them to obtain beneficial health knowledge and maintain a healthy lifestyle [37], so the role of education in promoting health is greater among rural residents than among urban residents.

##### Gender Differences in Education’s Effect on Health

As shown in Table 8, the gender difference in coefficients for education is statistically significant. Specifically, education has a greater role in promoting women’s health than men’s health. For each increase in the level of education, women’s self-reported health increases 0.024 grades more than men’s health, and the frequency at which women’s work is affected by health is 0.007 grades lower than the corresponding figure for men, both of which are significant at the level of 1%. Due to the influence of women’s reproductive roles and traditional beliefs, women tend to take more care responsibilities in the family [49], and this difference in the gender distribution of family care responsibilities has a great impact on gender inequality in the labour market. Women thus may face more “hidden discrimination” in the workplace [50] and enjoy many fewer economic and social resources than men. Education, as a relatively fair selection system, might play a vital role in promoting women’s personal abilities, advancing more development opportunities, and improving social and economic status. Therefore, education has a more significant role in promoting women’s health.

##### Age Differences in Education’s Effect on Health

The age differences in health promotion by education are shown in Table 9. Education has a significant effect on the subjective and objective health of the middle-aged and elderly groups, but it has no obvious effect on the health of the younger group of 39 years old and younger. This might be mainly because individuals in their youth are still relatively physically healthy, and most illnesses do not appear until middle and old age, so the effect of education on health is not obvious during this period. With increasing age, under the dual pressures of the workplace and the family, the individual’s loss of physical functions might also increases, and a series of health problems might occur. At this time, and especially in old age, the stronger economic capacity, healthier lifestyle and mental state due to a higher educational level might play a positive role in alleviating health problems and maintaining physical and mental health. This is also consistent with the findings of Cheng et al. [28].

##### Regional Differences in Education’s Effect on Health

As shown in Table 10, the role of education is greater in the western region than in the eastern and central regions. This result further confirms the resource substitution theory. Compared with the eastern region, the western region has relatively under-developed infrastructure, medical care, and economy [51]. The economic and social resources that residents could enjoy are relatively limited, and the education resource becomes an important way for residents to acquire health knowledge and cultivate good living habits, which helps promote physical and mental health. This result also shows that it is necessary to increase educational investment in the under-developed areas of the central and western regions. This could not only promote individuals’ educational level but also bring about a health premium for the whole society.

### 3.5. Robustness Test

To investigate the reliability of the above analysis, this section intends to test the robustness of the model. In fact, in the definition of the dependent variable, we use the subjective health evaluation and objective health level to confirm each other to avoid the bias caused by a single indicator. We reach a consistent conclusion in different tests, reflecting that the model has good stability. Here, we further investigate other dimensions. Since the two dependent variables in this article are ordered variables, the ordered logit model is used here to regress the data to test the robustness of the model. Moreover, we redefine the education level and divide it into four levels, assigning a value of 1 for elementary school and below (including uneducated, private school, literacy class, and primary school), 2 for junior high school, 3 for senior high school and equivalent education (including vocational school and technical school), and 4 for university and above (including junior college, undergraduate and postgraduate and above).

The ordered logit model implies the proportional odds assumption. So we test this assumption first, and the result shows that the assumption is violated, so we relax the assumption and use the Gologit model for regression. The results are shown in Table 11.

Table 11 shows that education has a significant positive correlation with the subjective health evaluation, which is reflected in Group 2 and Group 3. The higher the level of education is, the more self-reported health tends to shift from very unhealthy or relatively unhealthy to general, relatively healthy or very healthy (Group 2), and shift from very unhealthy, relatively unhealthy, general to relatively healthy or very healthy (Group 3), both of which are significant at the 1% level.

In addition, education has a significant negative correlation with the frequency of health effects on work. The higher the level of education is, the less often health affects work. For example, in Group 3 the frequency changes from regular or always to never, rarely, or sometimes. In Group 2, the frequency changes from regular, always, or sometimes to never or rarely, and in Group 1, the frequency changes from regular, always, sometimes, or rarely to never. All of these correlations are significant at the 1% level. For the other variables in the two models, the coefficients are basically consistent with the previous analysis in both direction and significance, indicating that the model has good robustness.

We also use the Gologit model to re-estimate the relationship between education and health with education as a dichotomous variable or seven-point variable, and we found the coefficients of most variables still keep consistent with the analysis of OLS model in both direction and significance (To save space, we did not report the estimation results here. Readers who need please contact us), which indicates that the main estimation results are robust.

## 4. Discussion 

The results show that education has a significant role in promoting the subjective and objective health of residents. Besides to directly promoting health, education is found to indirectly affects individual health through the three channels of enhancing mental status, economic status, and healthy behaviors. This is consistent with the explanation of “efficiency-improving effect”, “mental health effect”, and “budget relaxation effect”. However, considering the comprehensiveness of the questions asked on the survey and the availability of data, the constructs to the three effect that their composition and comprehensiveness may be limited. Further research finds that mental health plays a more important role than healthy behaviors and economic status. This may be because education increases personal economic income but also brings greater work intensity, such as working overtime, staying up late, and experiencing frequent business trips, which could reduce the time for personal leisure and other healthy activities, such as physical exercise, so it partially offsets the positive effect of improved economic status and healthy behavior.

In terms of specific channels, different channels reflect differences. Specifically, in the channel of mental health, education mainly improves individuals’ mental health by reducing the frequency of depression and improving well-being, but it has no obvious effect on enhancing social trust. In the channel of healthy behaviors, education mainly serves to increase the frequency of learning new knowledge and listening to music to promote the physical and mental health of individuals, but it has little effect on promoting physical exercise. In the channel of economic status, education mainly promotes personal health by improving the family’s economic status and the individual’s horizontal economic status. We also further analyzed the differences in the impact of education on health among different groups. The study finds that education more strongly improves the health of vulnerable groups with fewer available socioeconomic resources.

The findings of this article have the following important policy implications. First, since education is conducive to improving the health of residents, the government could pay more attention to the effect of education on health and adopt corresponding measures to take advantage of this effect. Specifically, it is strongly recommended that the government continue to promote compulsory education, develop various forms of vocational education, and expand the enrolment ratio of higher education to extend the possibility for individuals to improve their scientific and cultural literacy and accumulate human capital. Second, in the practice of education, educators could strengthen the teaching of physical and mental health knowledge, cultivate students’ positive and optimistic attitudes towards life to promote the comprehensive development of students and enable education to better promote health. Last, education has a more obvious role in promoting the health of vulnerable groups with fewer available resources, indicating that education could be an important supplement to solve the problems of a shortage of medical resources and under-developed medical facilities in disadvantaged areas. When the government allocates the public resources of education and health expenditures, it might be useful to prioritize education investment, especially in under-developed areas in the western region, where education still has a greater marginal effect on improving health. Such investment represents a favorable opportunity to guide education in promoting health. Increasing investment in education in this area could be a powerful way to mitigate the shortage of medical resources and improve residents’ health and human capital. 

## 5. Conclusions

Based on the CGSS2015 data, this article systematically investigates the relationship between education and individuals’ health status using the SEM, PSM, and Gologit model. We find that education has a significant role in promoting the subjective and objective health of residents, and this holds true after considering the issue of selection bias. Through further research, we find that in addition to directly promoting health, education indirectly affects individual health through the three channels of enhancing mental status, economic status, and healthy behaviors. This is consistent with the explanation of “efficiency-improving effect”, “mental health effect”, and “budget relaxation effect”. Further research finds that mental health plays a more important role than healthy behaviors and economic status.

Despite its contributions, this article also has some limitations. First, the CGSS2015 is cross-sectional data, which can only reflect the individual’s health status at a certain time and lacks the continuous tracking of samples over time, so it is impossible to investigate the possible changes in education’s impact on health over a longer time span. Second, limited by the availability of data, this article does not include more proximate determinants of health status already identified in the literature, such as lifestyle choices [52]. Thus, further research that includes these explanatory variables of health status is needed in order to assess education’ s relative importance to health more comprehensively. This article also does not include the unobservable ability factors that affect both education and health status in the model. Personal ability could promote education and health [43,53]. For example, people with stronger cognitive ability tend to have better academic performance, and people with stronger self-control tend to choose a healthy lifestyle and reduce bad habits, such as smoking, drinking, and staying up late. Thus, a model that does not include ability factors might overestimate the health promotion effect of education to a certain extent and entail certain errors. These limitations also point to potential directions worth further efforts in future research.

## Figures and Tables

**Table 1:** Descriptive statistics of variables.

Variable Type	Variable Name	Sample Size	Mean	Standard Deviation	Min	Max
Dependent variable	Self-reported health	8680	3.739	1.010	1	5
	Frequency of health effects on work	8680	2.019	0.978	1	5
Independent variable	Respondent’s education level	8680	3.253	1.451	1	7
Control variable	Gender	8680	0.478	0.500	0	1
	Age	8680	55.254	21.207	22	115
	Ethnicity	8680	0.924	0.265	0	1
	Political identity	8680	0.109	0.311	0	1
	Marital status	8680	0.791	0.406	0	1
	Father’s education level	8680	2.719	1.788	1	9
	Mother’s education level	8680	2.174	1.578	1	9
	Province dummy variable	28			0	1
Channel variable	Frequency of exercise	8680	2.518	1.517	1	5
	Frequency of learning	8680	2.035	1.095	1	5
	Frequency of listening to music	8680	2.503	1.481	1	5
	Happiness	8680	3.885	0.795	1	5
	Frequency of depression	8680	2.110	0.893	1	5
	Social trust	8680	3.470	0.959	1	5
	Horizontal economic status	8680	1.733	0.542	1	3
	Vertical economic status	8680	2.264	0.617	1	3
	Family economic status	8680	2.695	0.698	1	5

Source: CGSS2015.

**Table 2:** The effect of education level on health.

Variables	Health Evaluation			Frequency of Health Effects on Work		
	Model1	Model2	Model3	Model4	Model5	Model6
Education level	0.184 ***	0.133 ***	0.102 ***	−0.128 ***	−0.127 ***	−0.096 ***
	(0.007)	(0.008)	(0.010)	(0.005)	(0.008)	(0.010)
Age		−0.008 ***	−0.007 ***		0.008 ***	0.007 ***
		(0.001)	(0.001)		(0.001)	(0.001)
Ethnicity		−0.025	−0.020		−0.022	−0.028
		(0.047)	(0.047)		(0.048)	(0.047)
Gender		0.107 ***	0.118 ***		−0.106 ***	−0.117 ***
		(0.021)	(0.021)		(0.020)	(0.020)
Political identity		−0.043	−0.033		0.044	0.035
		(0.034)	(0.034)		(0.034)	(0.034)
Marital status		−0.000	0.019		−0.013	−0.032
		(0.025)	(0.026)		(0.024)	(0.024)
Father’s education			0.027 ***			−0.027 ***
			(0.008)			(0.008)
Mother’s education			0.033 ***			−0.031 ***
			(0.010)			(0.009)
Province	Yes	Yes	Yes	Yes	Yes	Yes
Observations	8680	8680	8680	8680	8680	8680
R-squared	0.101	0.129	0.133	0.102	0.128	0.133

Note: Standard errors in brackets, *** *p* < 0.01.

**Table 3:** The treatment effect of propensity score matching (PSM).

Dependent Variable	Matching Method	Average Treatment Effect	Standard Error	*t*-Test
	K-nearest neighbor matching (K = 4)	0.196 ***	0.071	2.75
**Self-Reported Health**	Caliper matching (Cal = 0.05)	0.220 ***	0.056	3.97
	Nearest neighbor matching within caliper (K = 4 Cal = 0.05)	0.196 ***	0.071	2.75
	Kernel matching Average value	0.224 *** 0.209	0.056	3.99
**The Frequency of Health Effects on Work**	K-nearest neighbor matching (K = 4)	−0.201 ***	0.069	−2.90
	Caliper matching (Cal = 0.05)	−0.218 ***	0.053	−4.07
	Nearest neighbor matching within caliper (K = 4 Cal = 0.05)	−0.201 ***	0.069	−2.90
	Kernel matching Average value	−0.217 *** −0.209	0.054	−4.01

Notes: *** *p* < 0.01.

**Table 4:** The data balancing test of PSM.

Variable	K-Nearest Neighbor Matching	Caliper Matching	Nearest Neighbor Matching within Caliper	Kernel Matching
Age	−75.5 (−5.3)	−75.5 (−11.2)	−75.5 (−5.3)	−75.5 (−10.8)
Political status	38.1 (17.9)	38.1 (17.4)	38.1 (17.9)	38.1 (17.1)
Gender	28.0 (−5.5)	28.0 (−0.9)	28.0 (−5.5)	28.0 (−1.1)
Ethnicity	13.8 (−4.8)	13.8 (−0.8)	13.8 (−4.8)	13.8 (−1.3)
Marital status	−16.6 (−18.5)	−16.6 (−19.8)	−16.6 (−18.5)	−16.6 (−19.6)
Father’s education	109.8 (−4.4)	109.8 (5.0)	109.8 (−4.4)	109.8 (4.2)
Mother’s education	106.8 (−4.5)	106.8 (7.9)	106.8 (−4.5)	106.8 (6.3)

Note: standardized deviations in brackets.

**Table 5:** The fit results of the structural equation modeling (SEM).

Indicator Type	Index	Critical Value	Model Result	Whether the Results Meet Requirement
Absolute fit index	RMSEA	≤0.08	0.067	Yes
	GFI	>0.9	0.963	Yes
	AGFI	>0.9	0.940	Yes
Relative fit index	NFI	>0.9	0.911	Yes
	IFI	>0.9	0.913	Yes
	CFI	>0.9	0.913	Yes
Simple fit index	PGFI	>0.5	0.593	Yes
	PNFI	>0.5	0.662	Yes
	PCFI	>0.5	0.664	Yes
	CMIN/DF	<2	39.722	No

Note: (1) RMSEA refers to the root mean squared error of approximation; (2) GFI refers to the goodness of fit index; (3) AGFI refers to the adjusted goodness of fit index; (4) CMIN/DF refers to the chi-square divided by degrees of freedom.

**Table 6:** Path coefficients of education’s impact on health.

Paths between Variables			Estimation				C.R.	Standard Errors
			Unstandardized Coefficient		Standardized Coefficient			
Health status	←	Education	0.050	***	0.099	***	5.042	0.010
Economic status	←	Education	0.063	***	0.253	***	17.698	0.004
Healthy behavior	←	Education	0.414	***	0.729	***	61.583	0.007
Mental health	←	Education	0.042	***	0.216	***	13.932	0.003
Health status	←	Economic status	0.114	***	0.056	***	4.599	0.025
Health status	←	Healthy behavior	0.102	***	0.114	***	5.390	0.019
Health status	←	Mental health	1.711	***	0.656	***	25.075	0.068
Exercise	←	Healthy behavior	0.846	***	0.459	***	35.353	0.024
Learning	←	Healthy behavior	1.000		0.751			
Listening to music	←	Healthy behavior	0.988	***	0.549	***	41.155	0.024
Social trust	←	Mental health	0.282	***	0.083	***	6.655	0.042
Depression	←	Mental health	−2.677	***	−0.847	***	−21.721	0.123
Sense of happiness	←	Mental health	1.000		0.356			
Horizontal economy	←	Economic status	1.000		0.668			
Vertical economy	←	Economic status	0.392	***	0.230	***	17.775	0.022
Family economy	←	Economic status	1.566	***	0.812	***	23.734	0.066
Health evaluation	←	Health status	1.000		0.734			
Work affected by health	←	Health status	−1.117	***	−0.847	***	−53.611	0.021

Note: *** *p* < 0.01; ← refers to a estimation path; C.R. refers to the critical ratio.

**Table 7:** Urban–rural differences in education’s effect on health.

Variables	Self-Reported Health			The Frequency of Health Effects on Work		
	Urban and Rural	Urban	Rural	Urban and Rural	Urban	Rural
Education	0.043 ***	0.033 ***	0.080 ***	−0.050 ***	−0.022	−0.079 ***
	(0.010)	(0.014)	(0.015)	(0.010)	(0.014)	(0.013)
Individual variables	Yes	Yes	Yes	Yes	Yes	Yes
Channel variables	Yes	Yes	Yes	Yes	Yes	Yes
Province	Yes	Yes	Yes	Yes	Yes	Yes
Sample size	8055	3282	4773	8055	3282	4773
R-squared	0.289	0.264	0.322	0.343	0.282	0.380

Note: Standard errors in brackets, *** *p* < 0.01.

**Table 8:** Gender differences in education’s effect on health.

Variables	Self-Reported Health			The Frequency of Health Effects on Work		
	All	Female	Male	All	Female	Male
Education	0.047 ***	0.056 ***	0.032 **	−0.053 ***	−0.055 ***	−0.048 ***
	(0.010)	(0.013)	(0.014)	(0.009)	(0.013)	(0.013)
Individual variables	Yes	Yes	Yes	Yes	Yes	Yes
Channel variables	Yes	Yes	Yes	Yes	Yes	Yes
Province	Yes	Yes	Yes	Yes	Yes	Yes
Sample size	8680	4534	4146	8680	4534	4146
R-squared	0.287	0.313	0.259	0.342	0.376	0.306

Note: Standard errors in brackets, ** *p* < 0.05, *** *p* < 0.01.

**Table 9:** Age differences in education’s effect on health.

Variables	Self-Reported Health			The Frequency of Health Effects on Work		
	≤39 Years Old	40–59 Years Old	≥60 Years Old	≤39 Years Old	40–59 Years Old	≥60 Years Old
Education	−0.017	0.034 **	0.027 *	−0.011	−0.049 ***	−0.045 ***
	(0.017)	(0.017)	(0.016)	(0.014)	(0.016)	(0.016)
Individual variables	Yes	Yes	Yes	Yes	Yes	Yes
Channel variables	Yes	Yes	Yes	Yes	Yes	Yes
Province	Yes	Yes	Yes	Yes	Yes	Yes
Sample size	2090	3379	3211	2090	3379	3211
R-squared	0.197	0.273	0.259	0.215	0.329	0.334

Note: Standard errors in brackets, * *p* < 0.1, ** *p* < 0.05, *** *p* < 0.01.

**Table 10:** Regional differences in education’s effect on health.

Variables	Self-Reported Health			The Frequency of Health Effects on Work		
	Eastern Area	Central Area	Western Area	Eastern Area	Central Area	Western Area
Education	0.004	0.067 ***	0.079 ***	−0.049 ***	−0.047 ***	−0.055 ***
	(0.013)	(0.019)	(0.021)	(0.012)	(0.018)	(0.019)
Individual variables	Yes	Yes	Yes	Yes	Yes	Yes
Channel variables	Yes	Yes	Yes	Yes	Yes	Yes
Sample size	4329	2255	2096	4329	2255	2096
R-squared	0.219	0.301	0.312	0.277	0.354	0.386

Note: Standard errors in brackets, *** *p* < 0.01.

**Table 11:** Robustness test of the health effects of education.

Dependent Variables	Group 1		Group 2		Group 3		Group 4	
	1 vs. 2, 3, 4, 5		1, 2 vs. 3, 4, 5		1, 2, 3 vs. 4, 5		1, 2, 3, 4 vs. 5	
	Subjective Health	Objective Health	Subjective Health	Objective Health	Subjective Health	Objective Health	Subjective Health	Objective Health
Education	0.106	−0.134 ***	0.210 ***	−0.183 ***	0.092 ***	−0.162 ***	−0.028	−0.010
	(0.122)	(0.033)	(0.049)	(0.039)	(0.033)	(0.060)	(0.035)	(0.139)
Age	−0.013 ***	0.012 ***	−0.014 ***	0.013 ***	−0.015 ***	0.016 ***	−0.019 ***	0.021 ***
	(0.005)	(0.001)	(0.002)	(0.001)	(0.001)	(0.002)	(0.002)	(0.005)
Gender	0.235	0.054	0.012	0.137	-0.118	−0.077	0.178 *	−0.210
	(0.261)	(0.098)	(0.121)	(0.105)	(0.094)	(0.141)	(0.107)	(0.302)
Ethnicity	0.142	−0.169 ***	0.138 *	−0.212 ***	0.231 ***	−0.106	0.137 **	0.214
	(0.173)	(0.052)	(0.071)	(0.058)	(0.051)	(0.087)	(0.055)	(0.204)
Political identity	−0.488	0.034	−0.250 *	0.274 ***	−0.363 ***	0.273 *	−0.025	0.208
	(0.308)	(0.085)	(0.132)	(0.100)	(0.087)	(0.159)	(0.091)	(0.376)
Marital status	0.033	−0.018	−0.096	0.104	−0.052	0.280 **	−0.145 **	0.133
	(0.205)	(0.066)	(0.090)	(0.075)	(0.065)	(0.114)	(0.069)	(0.262)
Father’s education	0.074	−0.079 ***	0.022	−0.035	−0.001	0.024	0.040 *	−0.031
	(0.072)	(0.021)	(0.031)	(0.025)	(0.021)	(0.036)	(0.022)	(0.090)
Mother’s education	0.025	−0.048 **	0.109 ***	−0.088 ***	0.068 ***	−0.198 ***	0.008	−0.339 **
	(0.081)	(0.023)	(0.039)	(0.030)	(0.025)	(0.050)	(0.024)	(0.142)
Horizontal economic status	−0.082	0.060	0.214 ***	−0.117 *	0.175 ***	−0.323 ***	−0.006	−0.021
	(0.189)	(0.057)	(0.074)	(0.063)	(0.055)	(0.093)	(0.061)	(0.216)
Vertical economic status	0.321 **	0.019	−0.070	0.012	0.096 **	0.104	0.071	0.082
	(0.125)	(0.043)	(0.056)	(0.047)	(0.042)	(0.068)	(0.045)	(0.144)
Family economic status	0.215	0.085 *	0.211 ***	−0.129 **	0.220 ***	−0.198 ***	0.137 ***	−0.168
	(0.143)	(0.045)	(0.059)	(0.051)	(0.044)	(0.074)	(0.048)	(0.160)
Exercise	0.056	0.023	0.004	0.004	−0.042 **	0.006	−0.028	−0.083
	(0.063)	(0.019)	(0.025)	(0.021)	(0.018)	(0.031)	(0.020)	(0.071)
Learning	0.138	0.019	0.151 ***	−0.025	0.118 ***	−0.104 *	0.077 **	−0.135
	(0.107)	(0.029)	(0.044)	(0.036)	(0.030)	(0.055)	(0.031)	(0.123)
Listening to music	−0.013	−0.104 ***	0.040	−0.071 ***	0.037 *	0.006	0.050 **	0.049
	(0.067)	(0.020)	(0.028)	(0.024)	(0.020)	(0.036)	(0.021)	(0.079)
Sense of happiness	0.201 **	−0.156 ***	0.115 ***	−0.077 *	0.189 ***	−0.009	0.385 ***	−0.219 **
	(0.091)	(0.038)	(0.044)	(0.040)	(0.036)	(0.053)	(0.043)	(0.103)
Social trust	0.119	0.064 **	0.080 **	0.038	0.020	−0.013	−0.056 *	0.042
	(0.080)	(0.027)	(0.036)	(0.031)	(0.027)	(0.044)	(0.029)	(0.094)
Depression	−1.299 ***	1.144 ***	−1.024 ***	1.493 ***	−0.839 ***	1.477 ***	−0.699 ***	1.440 ***
	(0.094)	(0.036)	(0.041)	(0.042)	(0.032)	(0.055)	(0.037)	(0.110)
N	8680	8680	8680	8680	8680	8680	8680	8680

Note: Standard errors in brackets, coefficients above the brackets, *** *p* < 0.01, ** *p* < 0.05, * *p* < 0.1.
